# Differences in synaptic vesicle pool behavior between male and female hippocampal cultured neurons

**DOI:** 10.1038/s41598-021-96846-y

**Published:** 2021-08-30

**Authors:** Sinem M. Sertel, Wiebke Blumenstein, Sunit Mandad, Orr Shomroni, Gabriela Salinas, Silvio O. Rizzoli

**Affiliations:** 1 Institute for Neuro- and Sensory Physiology, University Medical Center Göttingen, 37075 Göttingen, Germany; 2 Cluster of Excellence “Multiscale Bioimaging: From Molecular Machines to Networks of Excitable Cells” (MBExC), University of Göttingen, 37073 Göttingen, Germany; 3 NGS-Integrative Genomics Core Unit Göttingen (NIG), Institute of Human Genetics, University Medical Center Göttingen, 37077 Göttingen, Germany

**Keywords:** Molecular neuroscience, Neuronal physiology, Sexual behaviour, Synaptic plasticity, Synaptic transmission

## Abstract

A strong focus on sex-related differences has arisen recently in neurobiology, but most investigations focus on brain function in vivo, ignoring common experimental models like cultured neurons. A few studies have addressed morphological differences between male and female neurons in culture, but very few works focused on functional aspects, and especially on presynaptic function. To fill this gap, we studied here functional parameters of synaptic vesicle recycling in hippocampal cultures from male and female rats, which are a standard model system for many laboratories. We found that, although the total vesicle pools are similar, the recycling pool of male synapses was larger, and was more frequently used. This was in line with the observation that the male synapses engaged in stronger local translation. Nevertheless, the general network activity of the neurons was similar, and only small differences could be found when stimulating the cultures. We also found only limited differences in several other assays. We conclude that, albeit these cultures are similar in behavior, future studies of synapse behavior in culture should take the sex of the animals into account.

## Introduction

The primary hippocampal culture^1^ is one of the most common systems in neurobiology, having been used for more than 20,000 studies, ranging from fundamental investigations of neuronal morphology, synaptic function and disease^2–4^. These cultures are typically prepared from dissociated newborn rat hippocampi, from mixed male and female brains, without taking the sex of the animals into consideration. Since hippocampus function is known to have strong sex-dependent differences^5^, it is reasonable to ask whether neurons from female and male hippocampi also behave differently in the culture. This question is rendered all the more timely by the current science policies^6,7^, which enforce the use of both female and male samples in experiments.

Some evidence has already been obtained on differences between female and male-derived neuronal cultures. The activation of Gamma-aminobutyric acid (GABA) receptors in the hypothalamic cultures has been shown to be sex-dependent, even in the absence of gonadal hormones^8^. Similarly, the inhibitory effect of GABA receptors has been shown to develop later in male hippocampal cultures^9^. Another example of sex-dependent behaviors is that male neurons have been shown to have more elaborate dendritic arbors than female neurons in hippocampal cultures, when detailed morphological analyses were performed^10^ and they may also be more vulnerable to hypoxia than female neurons^11^, which might be influenced by sex-dependent intracellular calcium regulation^12^. Interestingly, even effects such as stimulation-induced excitotoxicity seem to be sex-dependent in culture^12^. Nevertheless, no systematic analysis of the function of such cultures has been performed, to our knowledge, and virtually no differences in presynaptic function or morphology have been uncovered between male and female cultures. To address this, we compared female and male neurons through experiments ranging from functional imaging to proteomics, focusing especially on synaptic vesicle recycling, which is the function on which most of the aspects of presynapse converge.

Neurotransmission release from the presynaptic compartment requires the fusion of neurotransmitter-filled synaptic vesicles to the plasma membrane, thereby setting free their contents of neurotransmitter molecules (exocytosis). The synaptic vesicle components are subsequently retrieved from the plasma membrane of the presynaptic neuron (endocytosis) and are turned into a new fusion-competent vesicle, in what is termed synaptic vesicle recycling^13^. Normally only a fraction of the vesicles engage in recycling under physiological activity, totaling ~ 50% of the vesicles in hippocampal cultures, with ~ 25% found on the surface membrane, in the form of a readily retrievable pool of vesicle membranes^14^, and another ~ 25% within the presynaptic boutons (e.g.^15^). The rest of the vesicles form a so-called reserve pool, whose involvement in functional reactions is unclear^16–18^, and whose function may be mostly related to collecting soluble cofactor proteins and providing them for exo- and endocytosis reactions^19–22^.

Sex-related differences are completely unknown for synaptic vesicle pools, especially as these pools can be studied most effectively in cultured neurons, where sex-related issues have been only little investigated so far. Here we used several well-established tools to investigate vesicle pools, and found that they are different between male and female neurons, at least for hippocampal neurons of ~ 15 days in vitro, which are the most common culture model for synapse investigations. The total vesicle pools were similar, but the recycling pool was larger for synapses in male neurons, and was more frequently used. This correlated well with the fact that local translation, which has been often connected to synaptic function^23^, was higher in synapses of male neurons.

## Results and discussion

We prepared cultures from the hippocampi of newborn male and female rats^24^, separately, and allowed them to mature and to establish synaptic connections until at least *day *in vitro (DIV) 15. The cultures had similar cell numbers, and similar proportions of neurons and glia (Supplementary Figure S1). The overall morphology, which has been analyzed in detail in the past^10^, appeared similar, and the cellular volumes were similar (Supplementary Figure S2).

**Figure 1:**
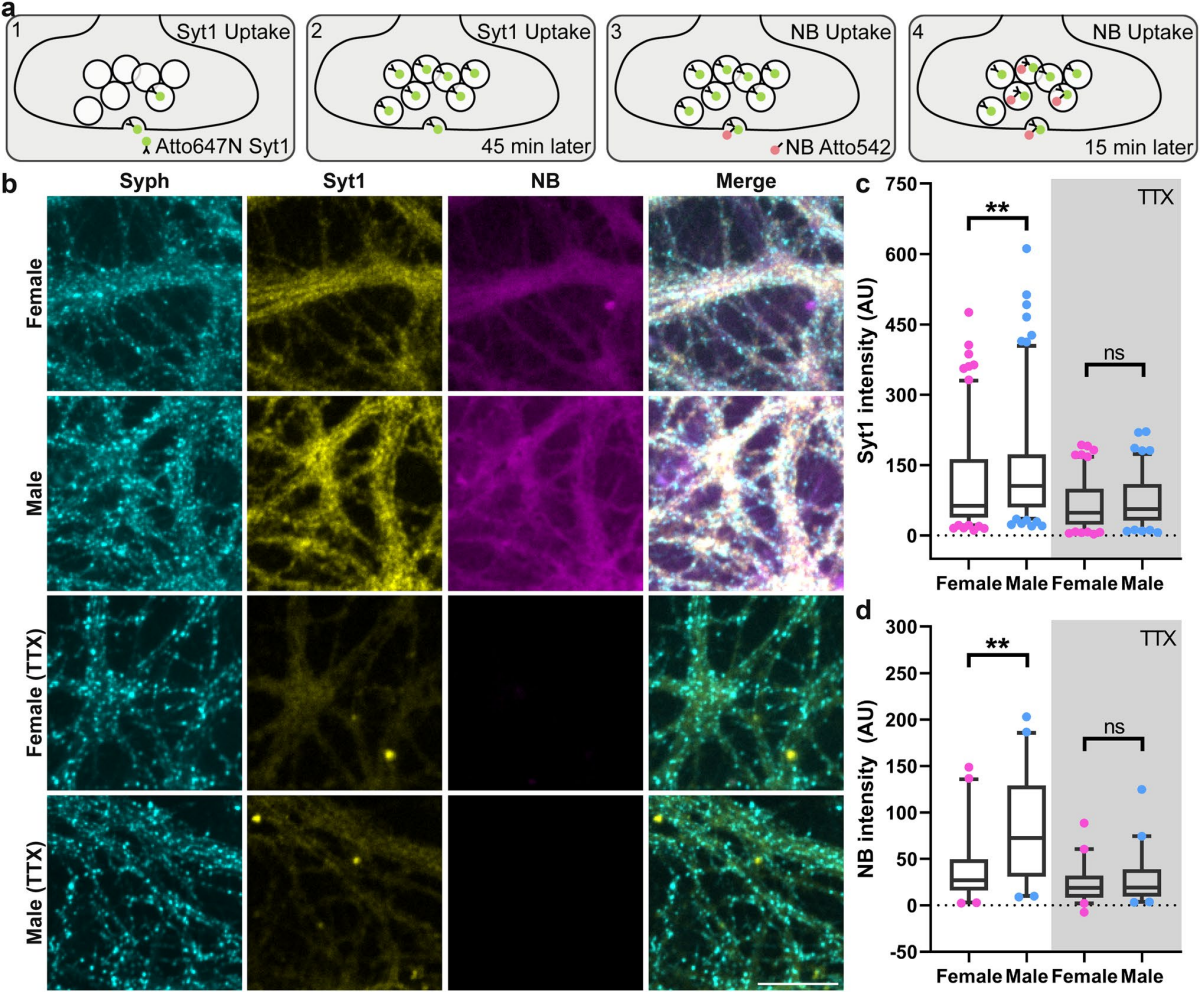
Male hippocampal neurons have a higher synaptic activity. (**a**) To study synaptic activity, we incubated the neurons for 45 min with an Atto647N-conjugated synaptotagmin 1 (Syt1) antibody (1). The intravesicular domain of Syt1 was detected by the antibody during the synaptic vesicle recycling (2). To estimate synaptic activity, we then added secondary nanobodies (NB), which detect the Syt1, and incubated for 15 min (3). The NB labeled vesicles represents the synaptic activity level during the respective 15 min (4). The cultures were immunostained for a synaptic marker (Syph) To label only the surface epitopes^12^, we stopped action potential generation with a tetrodotoxin (TTX) treatment. (**b**) Exemplary images of female and male neurons, including the TTX treatment. Scale bar: 50 µm. **(c)** To compare the recycling vesicle pool sizes in male and female neurons, we measured the intensity of the Syt1 antibody in Syph positive areas. The box plots show the first and third quartiles, as well as the median of the distribution. The whiskers indicate the 5–95 percentile. The shown symbols are the outliers. Each symbol represents the value measured from a whole Syt1 image. The statistical comparison between the two sexes was performed by the Kruskal–Wallis test together with the Dunn’s multiple comparisons test. **p < 0.01. N = 7 independent experiments, with 5 images taken for each experiment. **(d)** To compare the synaptic activity, we measured the NB intensity in the presynaptic area. The box plots and symbols are similar to those in panel c. **p < 0.01. N = 4 independent experiments, with 5 images taken for each experiment.

To determine functional differences between these cultures, we first compared their synaptic activity, relying on a well-established assay, in which the intravesicular domain of synaptotagmin 1 (Syt1) is detected by fluorescently-conjugated antibodies (Fig. 1a), which are then endocytosed^25,26^. A 45-min incubation with the antibodies is sufficient to saturate the entire pool of recycling vesicles^15^, thereby providing a measure of its size. To also provide a measure of the activity of the synapses, we added a fluorescently-conjugated nanobody that detects the antibodies^27^ for 15 min. The nanobody detects the vesicles that recycle during the respective 15 min, and thus provides a direct measurement of synaptic exo- and endocytosis.

Both labels could be detected readily in the cultures (Fig. 1b), and in both cases the male neurons showed significantly higher intensities (Fig. 1c,d). Blocking synaptic activity with the Na^+^ channel blocker tetrodotoxin (TTX), which halts the action potential generation, enabled us to test differences between the spontaneously recycling pools of vesicles^28^. As expected^15^, this treatment strongly reduced the labeling. At the same time, it removed any differences between the two types of cultures, suggesting that, as for the total vesicle pools, there is little difference between the spontaneously recycling pools of male and female neurons.

To test the surface pool of vesicle molecules, we performed this assay while keeping the neurons on ice. This only enables the detection of the surface pool of Syt1 molecules, which are representative for the surface vesicle pool^14,15^. This resulted in a low labeling that was similar for male and female cultures (Supplementary Figure S3).

**Figure 2:**
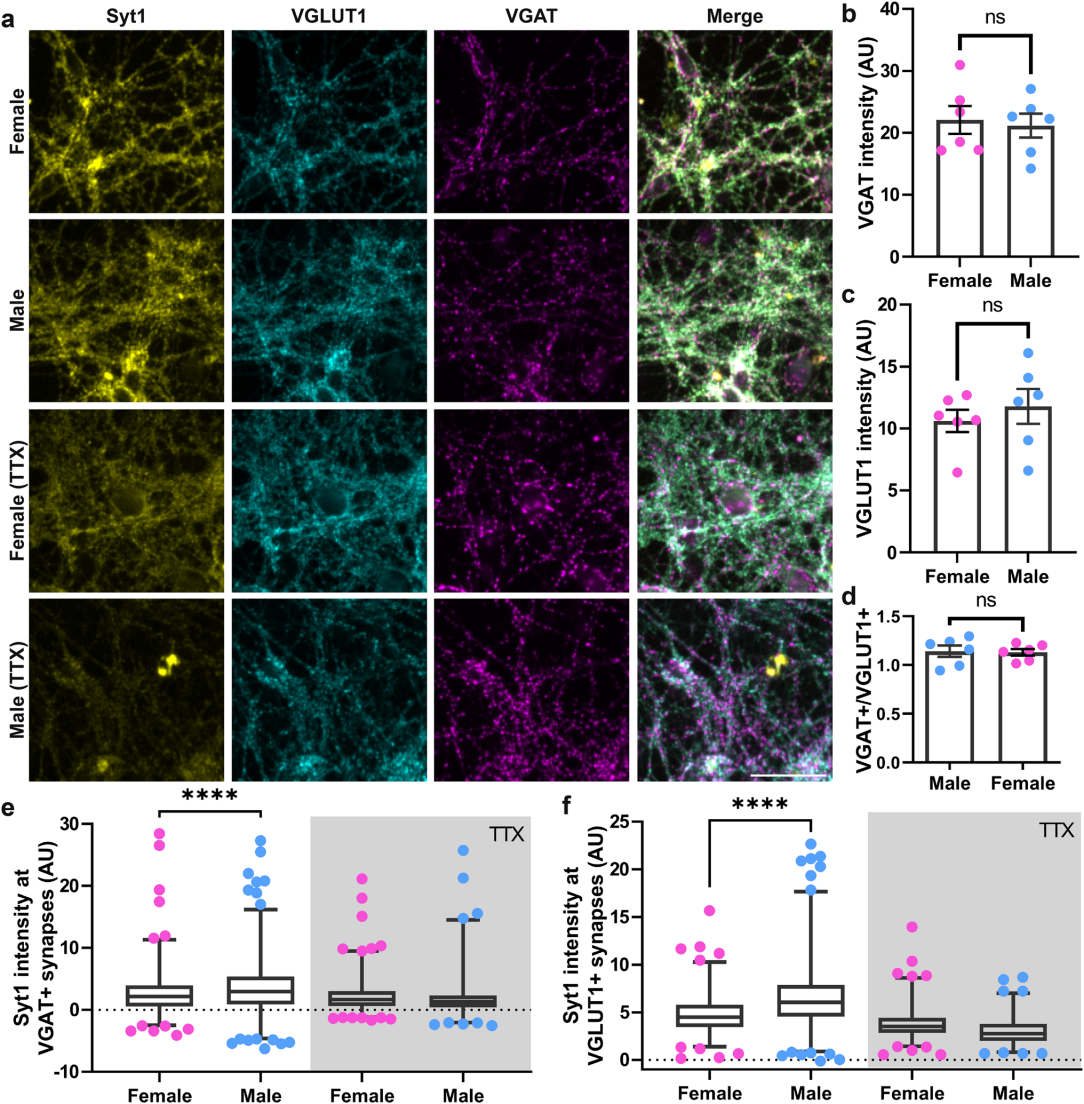
The inhibitory and excitatory synapses in male cultures have a higher activity than in female cultures. (**a**) To investigate the sex-dependent differences in inhibitory and excitatory synapses, we incubated the neurons, as in Fig. 1, for 45 min with an Atto647N-conjugated Syt1 antibody. The cultures were then immunostained with antibodies for two markers of inhibitory and excitatory synapses, the neurotransmitter transporters VGLUT1 (glutamate transporter) and VGAT (GABA transporter). Similar to Fig. 1, we used a TTX treatment as a control. Exemplary images of female and male neurons, including the TTX treatment, are shown. Scale bar: 50 µm. **(b)** and **(c)** We first analyzed the VGLUT1 and VGAT intensities in the two cultures. No significant differences could be detected (Mann–Whitney ranksum tests). **(d)** To test whether the proportion of inhibitory and excitatory synapses is different in the two cultures, we checked the ratio of VGAT positive (VGAT+) to VGLUT1 positive (VGLUT1+) synapses. No significant differences could be detected (Mann–Whitney ranksum test). For b, c and d panels, dots show the median of an image. The bar graph represents the mean ± SEM. N = 6 independent experiments and 5 images were taken per condition. We also analyzed the density of the VGLUT1 and VGAT spots. No differences could be found. The density of VGLUT1 spots in male cultures was 122 ± 27% of that in female cultures; the value was 115 ± 18% for male cultures (*p* > 0.48, Mann–Whitney ranksum tests; N = 6 independent experiments). **(e)** and **(f)** To compare the synaptic recycling pool in VGAT+ and VGLUT1+ synapses, we measured the Syt1 antibody intensity in the VGAT or VGLUT1 positive areas. The box plots show the first and third quartiles, as well as the median of the distribution. The whiskers indicate the 5–95 percentile. Symbols show the outliers. Each symbol represents the value obtained from a whole Syt1 image. The statistical comparison between the two sexes was performed by the Kruskal–Wallis tests, together with the Dunn’s multiple comparisons test. ****p < 0.0001. N = 3 independent experiments, with 5 images taken for each experiment.

To investigate the synaptic activity in more detail, we performed the same assay using excitatory and inhibitory synaptic markers, VGAT (Vesicular Gamma Aminobutyric Acid Transporter) and VGLUT1 (Vesicular Glutamate Transporter 1), respectively (Fig. 2a). We did not find any differences between the VGAT (Fig. 2b) or VGLUT1 (Fig. 2c) abundances in the male and female cultures. The culture network also contains similar amounts of inhibitory and excitatory synapses (Fig. 2d). However, the synaptic vesicles were recycled again more in male cultures, regardless of the synapse type (Fig. 2e,f).

Overall, these results suggest that the main difference between male and female neurons, in terms of vesicle pools, is the size and usage of the recycling pool, suggesting that male neurons use their vesicles more often. As activity strongly correlates with the synaptic turnover^29^, we then tested whether the sex-related activity differences were mirrored by differences in local turnover. We first used a metabolic labeling assay^30^, in which the cultures were incubated with a methionine substitute, L-Homopropargylglycine (HPG), for 4 h. The HPG molecules become incorporated in the newly-synthesized proteins, in the place of methionine, and can be then revealed by click chemistry (Supplementary Figure S4a). No differences could be found, either in synapses or elsewhere (Supplementary Figure S4b-e), implying that protein production (and presumably turnover) is overall similar in the male and female cells.

**Figure 3:**
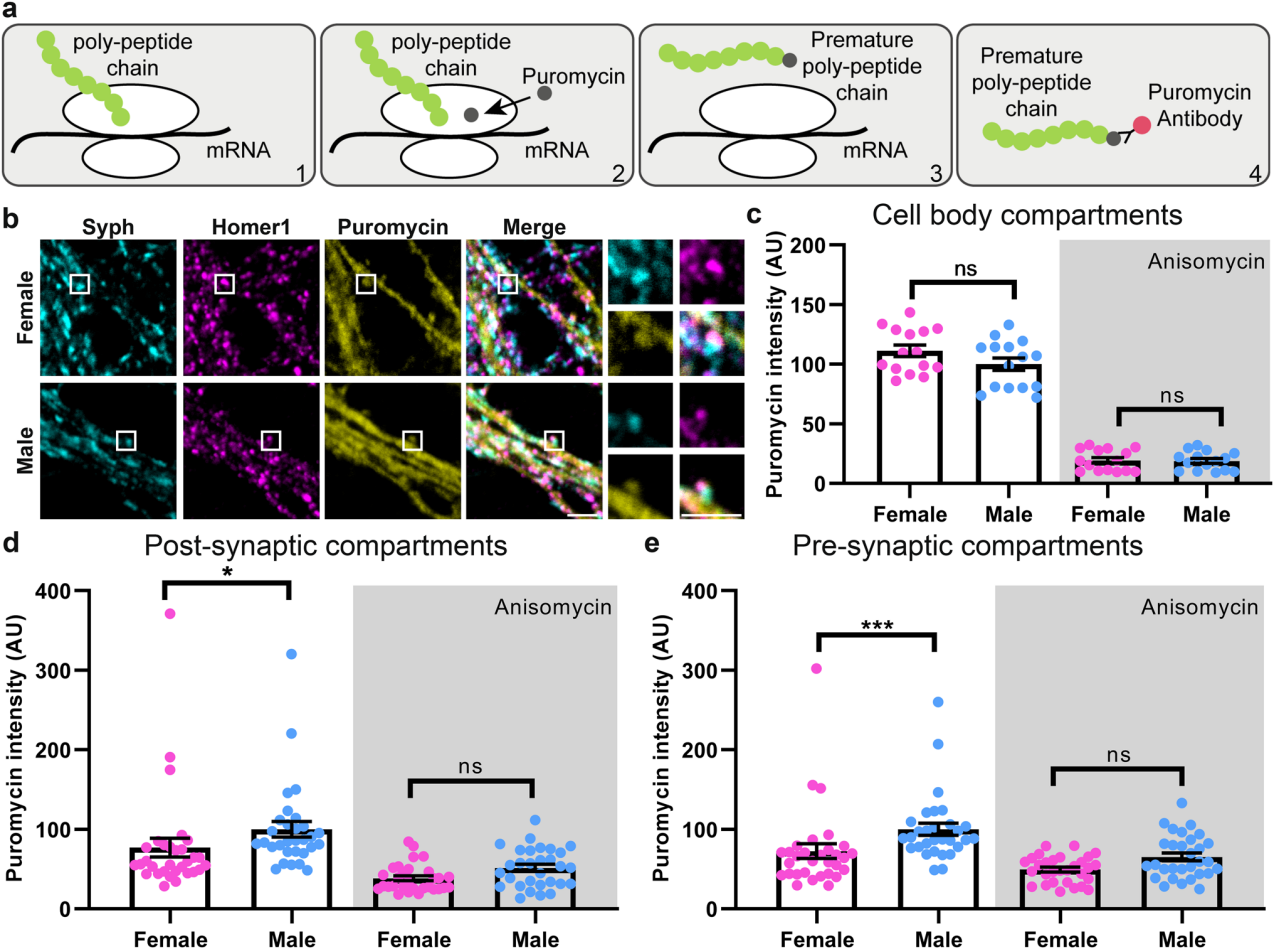
Male hippocampal neurons have a higher synaptic translation rate. (**a**) To report local translation rates, we performed a “puromycin assay”. During translation, the ribosome moves along the mRNA, enabling the growth of the poly-peptide chain (1). Puromycin interferes with the translation by binding to the ribosome, and stops the translation by incorporating itself into the premature polypeptide chain (2). It will release the premature poly-peptide chain (3). An immunostaining against puromycin shows the amount of translation at a certain location (4). To calculate the background of the puromycin treatment, we used another antibiotic, anisomycin, which prevents the incorporation of puromycin to a poly-peptide chain (not shown in the cartoon). (**b**) Exemplary images for the puromycin assay, along with Syph and Homer1 immunostainings, which indicate the pre- and postsynaptic sites. Scale bar: 2.5 µm. (**c**),(**d**) and (**e**) The intensity of puromycin staining was measured in the cell body, post- and presynaptic compartments. Dots represent the mean of an image. The bar graphs indicate the mean ± SEM. N = 4 independent experiments, for panel c 5 images and for panels d and e 10 images were taken per condition. The statistical comparison was performed by the Kruskal–Wallis test together with the Dunn’s multiple comparisons test. *p < 0.05, ***p < 0.001. Male neurons show significantly higher translation rate at the synapse, but not in the cell body.

To follow up on these findings, we compared the protein production within the synaptic compartments^23^. We performed a similar assay, in which the cells were incubated with the antibiotic puromycin, which incorporates itself into the growing polypeptide chain during translation, and releases it from the ribosome (Fig. 3a). This assay indicates with high specificity the synthesis of new protein chains in the synapses (as opposed to their transport to synapses from other locations, which cannot be excluded in the case of HPG labeling). We found that puromycin labeling was similar for the male and female cell bodies, but was significantly higher for the male synapses, for both the pre- and postsynaptic sides (Fig. 3b–e).

**Figure 4:**
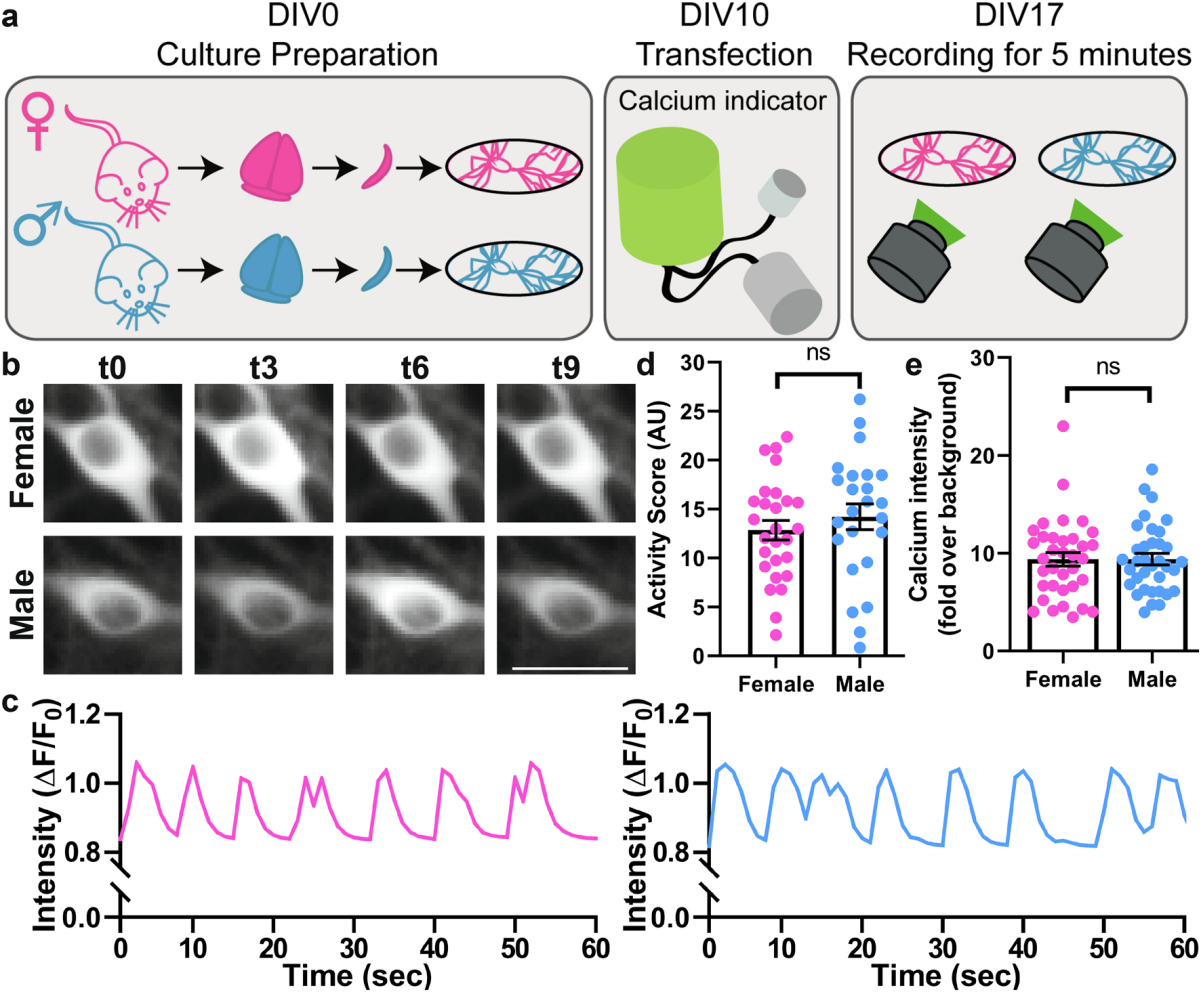
The spontaneous network activity does not show any sex-related differences. **(a)** To be able to study the sex-related differences in primary culture, we separated female and male neurons during the culture preparation. To determine the spontaneous firing rate, we transfected the neurons at day in vitro 10 (DIV10) with the genetically-encoded Ca^2+^ indicator NeuroBurst. At DIV17, we imaged neurons for 5 min. **(b)** Exemplary 9 s-long calcium signals (t0-t9) images of female and male neurons. Scale bar: 20 µm. **(c)** Example calcium signals in male and female neurons, normalized to the baseline (ΔF/F0). **(d)** To compare the spontaneous calcium activity between the two sexes, we calculated the area under the curve of the intensity graphs, which we termed “activity score”, over 6 independent culture preparations that have 6 coverslips for each sex (N = 6, n = 36 independent experiments). The graph presents the activity score (mean ± SEM) and each symbol represents the mean activity score of a coverslip. Maximum 10 neurons were selected per coverslip. The statistical comparison between the two sexes was performed by the Mann–Whitney test, not significant (ns). **(e)** To examine the NeuroBurst expression levels, we calculated the fluorescence intensity in the soma versus the background. The graph presents the baseline intensity over background (mean ± SEM) and each dot indicates the mean of a coverslip. The statistical comparison between the two sexes was performed by the unpaired t-test, not significant (ns).

The functional differences in synaptic activity, as well as translation differences, pointed out possible differences in the spontaneous firing rate. To take a closer look at the calcium activity, we relied on Ca^2+^ imaging. We transfected the cultures at DIV10 with a genetically-encoded Ca^2+^ indicator, NeuroBurst (Fig. 4a). At DIV17 we imaged the neurons to determine their spontaneous activity patterns, focusing on the evident Ca^2+^ dynamics in the somas (Fig. 4b). Both the frequency and the intensity of the activity events appeared similar (Fig. 4c). For a more thorough comparison, we measured the areas under the peaks of the normalized calcium intensity curves, which provides a summed “activity score” for each cell^31^. This measurement suggests that female and male neurons exhibit similar base-line NeuroBurst expression as well as similar levels of spontaneous activity (Fig. 4d,e). Furthermore, we analyzed several parameters of the calcium signals, such as event frequency, length and intensity (Supplementary Figure S5), without finding any significant differences between the two sexes.

**Figure 5:**
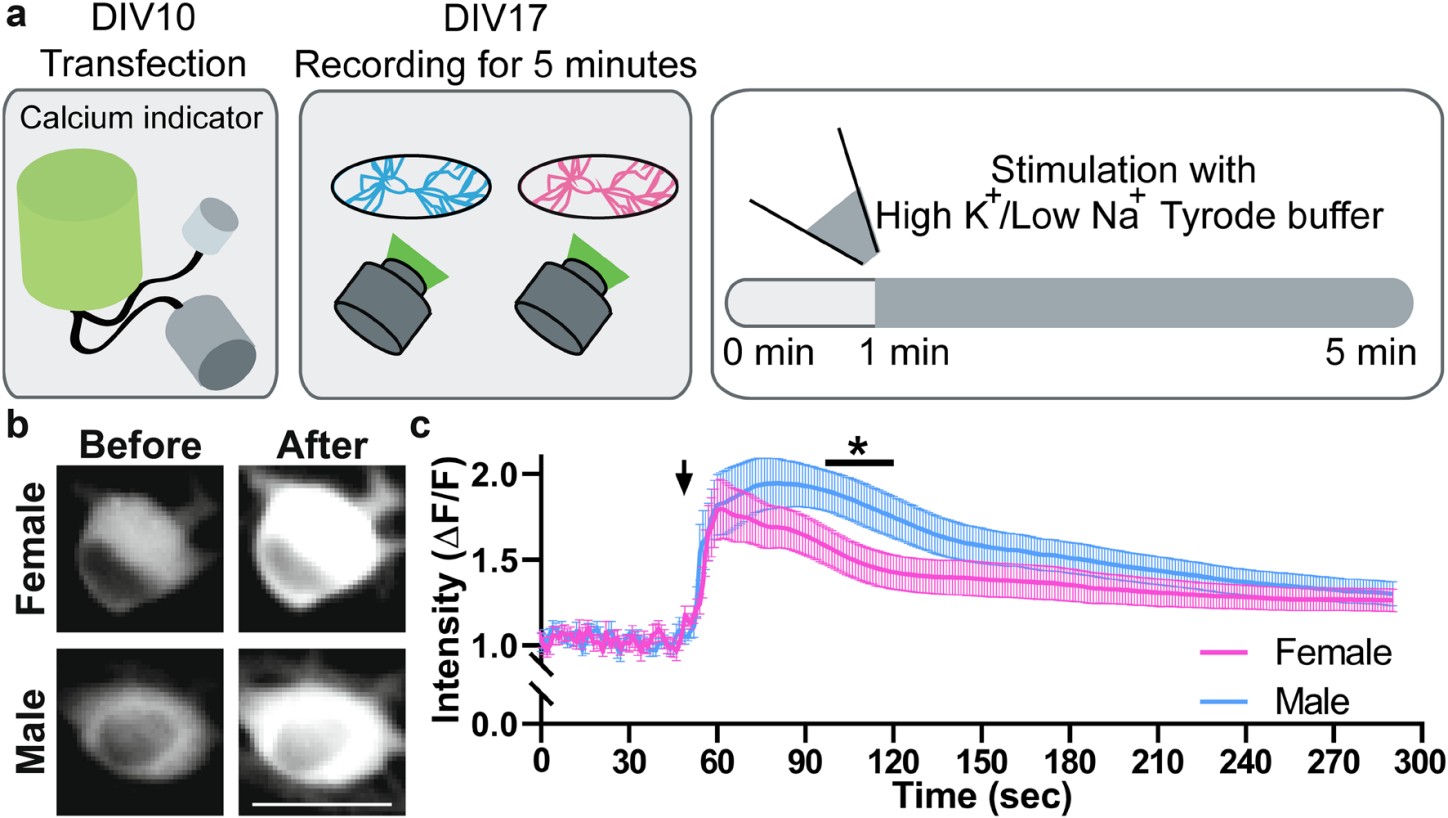
Male hippocampal neurons respond more strongly to prolonged depolarization. (**a)** Similar to Fig. 3, we performed calcium imaging. As an addition, we stimulated the culture with high potassium/low sodium Tyrode buffer at the 1-min mark to determine the response to a non-physiological stimulation. **(b)** Example images before and after the stimulation are shown. Scale bar: 20 µm. **(c)** The calcium intensities are plotted as the mean (± SEM) of female and male coverslips, with the arrow indicating the time point of the stimulation. We monitored 3 independent cultures, using 6 coverslips for each sex (N = 3, n = 18). Maximum 10 neurons were selected per coverslip. The statistical comparison was performed with the two-way ANOVA test. * p < 0.05. The bar indicates time points where the statistical test was showing significant differences.

Therefore, the “physiological” activity of the neurons seems to be similar for both male and female cultures. To verify their capacity to respond to stimuli that surpass the physiological levels, we subjected the cells to a prolonged depolarization, using a high K^+^/low Na^+^ buffer (Fig. 5a). The peak Ca^2+^ increase was similar for both male and female cultures, but the male cultures took longer to reach a Ca^2+^ plateau, possibly due to differences in the speed of the Ca^2^^+^ channel inactivation^32^, or to differences in Ca^2+^ buffering dynamics (Fig. 5b,c). The dynamics observed here are typical for such prolonged high K^+^ stimulation^33–35^.

To study the neuronal dynamics by a different method, we then used a voltage-sensitive membrane dye (FluoVolt, Thermo Fisher) (Supplementary Figure S6). The recordings were performed in a similar fashion to the Ca^2+^ imaging from Fig. 5. However, instead of chemical stimulation, we stimulated cultures electrically, applying long square pulses. Neither the spontaneous activity (Supplementary Figure S6a-j), nor the electrical stimulation (Supplementary Figure S6k-l) showed any differences between the two sexes.

These observations suggest that male neurons contain larger pools of active vesicles, and are more active, at the single-synapse level. To test whether this relates to different levels of key synaptic proteins, we immunostained vesicle markers (VAMP2, synaptophysin, VGLUT1), active zone markers (bassoon), cytoskeletal components (tubulin, MAP2), postsynaptic receptors (GluR2, Glun2B (Glun2A domain was substantially less abundant in the culture and therefore not selected for the survey)), postsynaptic density components (PSD95, Homer1), important synaptic regulators (α-synuclein, RBM3) and calcium binding proteins (calbindin, calmodulin and calreticulin). Only three proteins showed different intensities, bassoon, synaptophysin and calbindin (Supplementary Figure S7), and the overall differences were small. The number of objects (such as synapses, active zones, PSDs) was similar for all investigated proteins (Supplementary Figure S8). Cultures from other regions, such as cortex and hypothalamus, do show sex-dependent differences in the abundance of calbindin^36,37^. These small differences in protein abundance might have a contribution to the intracellular calcium regulation, and then to calcium dynamics and synaptic activity.

To confirm these observations at the whole-culture level, we proceeded with—omics analysis at transcript (Supplementary Figure S9a,b) and protein (Supplementary Figure S9c) levels. Beside the expected differences in Y-chromosome linked transcripts, we only found a slight tendency for higher amounts of transcripts relating to neuronal function in the female cultures (Supplementary Figure S9b), although this trend did not propagate to the protein amounts (Supplementary Figure S9c). Only small portion of the annotated transcripts and proteins are enriched in one of the cultures (Supplementary Figure S9d), and the mutual hits between these two datasets are very few (only a handful, shown in Supplementary Table S1). This implies that a gene enrichment or network analysis is difficult to perform on these data.

Overall, these observations lead to the conclusion that male synapses have larger recycling vesicle pools, which they employ more often than female synapses. The male neurons also d engage in higher levels of local translation, although this does not result in differences at the level of network activity, presumably due to compensatory mechanisms. One such possible mechanism would be higher synaptic activity in both excitatory and inhibitory neurons in male cultures, which thereby leads to similar overall network firing levels, albeit many other scenarios could be envisioned.

Our observations lead to a two-pronged conclusion. First, the limited differences noted here imply that most experiments using cultures formed of mixed male and female neurons are likely to be unaffected by sex-related differences, which is an important finding for the field. Second, the significant differences found for synaptic activity and local translation imply that studies of synapses should take the animal sex into account, and should employ the same numbers of female and male animals for each culture preparation, to ensure adequate reproducibility.

## Methods

### Animals

Rats (*Rattus norvegicus*. wild type, Wistar) for the primary neuron cultures were obtained from the animal facility of the University Medical Center Göttingen. Animals were handled according to the specifications of the University of Göttingen and of the local authority, the State of Lower Saxony (Landesamt für Verbraucherschutz, LAVES, Braunschweig, Germany). All experiments and procedures were approved by the local authority, the Lower Saxony State Office for Consumer Protection and Food Safety (Niedersächsisches Landesamt für Verbraucherschutz und Lebensmittelsicherheit). All methods are reported in accordance with ARRIVE guidelines and were carried out in accordance with relevant guidelines and regulations. The experimental procedure was approved by the relevant institutional entity, the Tierschutzbüro of the University Medical Center Göttingen (approval number T 09/08).

### Hippocampal cultures

Newborn female and male rats were sacrificed for the primary dissociated hippocampal culture preparation^24^. The sex determination was performed by animal facility personnel according to the animal morphology^38^. Hippocampi were dissected and washed in HBSS (Thermo Fisher, US). For dissociation, dissected hippocampi were incubated for 1 h with the enzyme (1.6 mM cysteine, 100 mM CaCl_2_, 50 mM EDTA, and 25 units papain in 10 ml DMEM (Thermo Fisher, US)). 5 ml of DMEM, containing 10% fetal calf serum (FCS), 0.5% albumin, and 0.5% trypsin inhibitor, was used for enzyme inactivation. After an enzymatic treatment, cells were dissociated further by pipetting. 80,000 neurons were plated on 1.8 cm in diameter glass coverslips, which were coated with poly-L-lysine (PLL, Sigma-Aldrich, Germany). Cultures were incubated at 37 °C for one hour with a plating medium which is 3.3 mM glucose, 2 mM glutamine, and 10% horse serum in DMEM. Media were replaced with Neurobasal-A phenol-red free medium (Thermo Fisher, US), containing a B27 supplement (Thermo Fisher, US), 1% GlutaMax (Thermo Fisher, US), and 0.2% penicillin and streptomycin mixed solution. Until the use in experiments, cells were incubated at 37 °C and 5% CO_2_. The experiments on female and male cultures were always performed in parallel.

### Immunostaining

Before fixation, cultures were washed with Tyrode buffer which is 124 mM NaCl, 5 mM KCl, 2 mM CaCl_2_, 1 mM MgCl_2_, 30 mM D-glucose, and 25 mM HEPES. They were fixed for 30 min at room temperature with 4% Paraformaldehyde (PFA, Sigma-Aldrich, Germany). To quench the coverslips, they were incubated at room temperature for 30 min in 100 mM NH_4_Cl in phosphate buffer solution (PBS). Later, cells were washed three times with Triton-X-100 solution (Permeabilization solution, 3% bovine serum albumin (BSA), 1:10,000 Triton-X-100 in PBS). This was followed by an 1-h incubation with the primary antibody (in the permeabilization solution, see the Supplementary Table S2). Again cells were washed three times and stained with the secondary antibody (in the permeabilization solution, see the Supplementary Table S2) for 1 h. If it is specified, to visualize nuclei, cells were also stained with the Hoechst dye (Thermo Fisher, US, 1:1000 ratio in PBS). The antibodies are listed in the Supplementary Table S2. Coverslips were mounted with Mowiol (Merck Millipore, Germany). Lastly, they were stored in the fridge (4 °C). For imaging, different microscopes were used. Unless it is specified, a confocal microscope (Abberior, Germany) built on an IX83 inverted Olympus microscope body (Japan) was used. The set-up has a 100X super-apochromat and coverslip corrected oil objective (Olympus, Japan). A LSM 780 laser scanning confocal microscope (Zeiss, Germany), with an Examiner Z1 microscope body (Zeiss, Germany), was used for the immunostaining survey on synaptic proteins, using a 20X water objective (Plan-apochromat, Zeiss, Germany) and an AxioCam camera (Zeiss, Germany).

### Calcium imaging

NeuroBurst Orange Lentivirus (Sartorius, Germany) was used as a genetically-encoded calcium indicator. Virus transduction was performed on day in vitro (DIV) 10 with 3 µl of NeuroBurst. On DIV17, glass coverslips were prepared in an imaging chamber with Tyrode buffer (400 µl) and imaged at 37 °C with an inverted Nikon Ti eclipse epifluorescence microscope (Nikon, Japan). This set-up has an HBO-100 W lamp, a 20X Plan Apo (Nikon, Japan) objective, an IXON X3897 camera (Andor, UK), and an Okolab cage-incubator (Italy). Stimulation was performed with the addition of 400 µl high K^+^/low Na^+^ Tyrode buffer (70 mM KCl, 59 mM NaCl, 2 mM CaCl_2_, 1 mM Mg_2_Cl, 30 mM D-Glucose and 25 mM HEPES) on top of the initial 400 µl of Tyrode buffer. This solution was not removed till the end of the recording.

### Synaptotagmin1 (Syt1) uptake assay

This assay performed as described before^31^. Briefly, neurons were incubated for 45 min with in a Neurobasal-A medium with 8.3 μM Syt1 antibody (105311AT1, Synaptic Systems, Germany). This step was followed by the addition of anti-mouse secondary nanobody (16.7 nM, N2002-At542-S, Nanotag, Germany). Cells were washed 15 min later with Tyrode buffer. After fixation with 4% Paraformaldehyde (PFA), cells were immunostained with Synaptophysin (Syph) antibody (101,004, Synaptic Systems, Germany). As a control, a Na^+^ channel blocker tetrodotoxin (TTX, Tocris Bioscience, UK) was used. The coverslips were imaged with the Nikon microscope which is described in the previous section.

### Puromycin assay

1 µg/ml of puromycin (ant-pr-1, InvivoGen, US) was added into the cultures. After 10 min of incubation, coverslips were washed with Tyrode buffer. The fixation was followed with 4% PFA. Anisomycin (0.13 μM, A5862, Sigma-Aldrich, Germany) was used in order to detect the background signal of the assay. For the control group, it was added 10 min before the actual puromycin treatment. After the fixation, cells were immunostained, as described in the immunostaining section, against Synaptophysin (101004, Synaptic Systems, Germany), Homer1 (160011, Synaptic Systems, Germany), and puromycin (MABE343, Merck Millipore, Germany).

### Image analysis

Matlab (MathWorks, US) was used for image analysis, and plots were prepared in GraphPad Prism version 8.00 (GraphPad Software, US). All analysis was performed using simple thresholding procedures, based on empirically-derived thresholds, followed by the analysis of signal intensities within the threshold-defined ROIs. The ROIs were defined in the synaptic marker channels (Syph, Homer 1) for synaptic experiments, and not in the channels representing the measurements of interest (Syt1, puromycin, HPG).

## Supplementary Information

Supplementary Information 1.

Supplementary Information 2.
