# Supplementary material for: Differences in synaptic vesicle pool behavior between male and female hippocampal cultured neurons
Source: Sci Rep. 2021 Aug 30;11:17374. doi: 10.1038/s41598-021-96846-y (PMC8405817; doi:10.1038/s41598-021-96846-y)
Supplement: Supplementary file 1 — Supplementary Information 1. [file 41598_2021_96846_MOESM1_ESM.docx]

**Supplementary information for** *Differences in synaptic vesicle pool behavior between male and female hippocampal cultured neurons*

Sinem M. Sertel^1,2,*^, Wiebke Blumenstein^1,2^, Sunit Mandad^1,2^, Orr Shomroni^3^, Gabriela Salinas^3^, Silvio O. Rizzoli^1,2,4,*^

^1^Institute for Neuro- and Sensory Physiology, University Medical Center Göttingen, Göttingen, 37075, Germany

^2^Cluster of Excellence "Multiscale Bioimaging: from Molecular Machines to Networks of Excitable Cells" (MBExC), University of Göttingen, Göttingen 37073, Germany

^3^NGS-Integrative Genomics Core Unit Göttingen (NIG), Institute of Human Genetics, University Medical Center Göttingen, Göttingen, 37077, Germany

^4^Lead Contact

*Correspondence: [sinem.sertel@med.uni-goettingen.de](mailto:sinem.sertel@med.uni-goettingen.de) (S.M.S.), [srizzol@gwdg.de](mailto:srizzol@gwdg.de) (S.O.R.)

This file contains the following sections:

- Supplementary Figures S1-9
- Supplementary Table S1
- Supplementary Methods
- Supplementary information


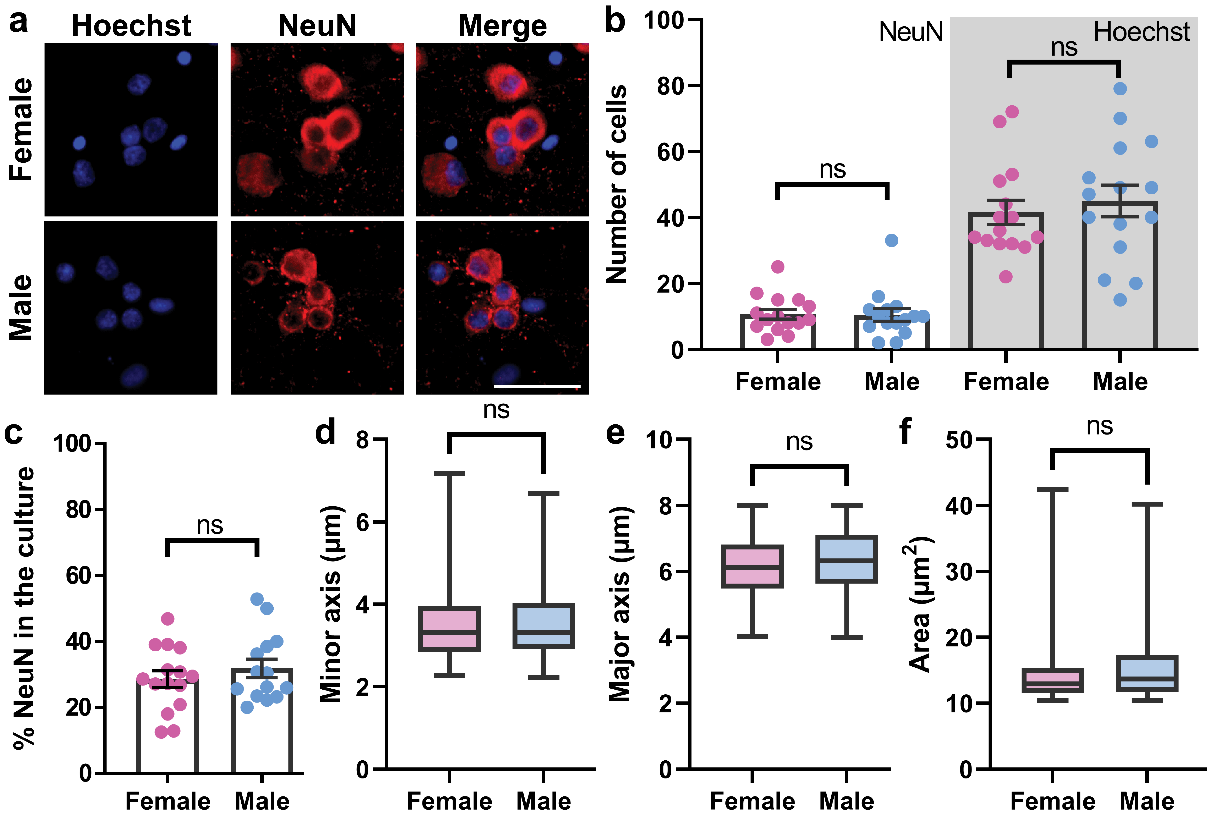


**Supplementary Figure S1. Female and male hippocampal neurons have the same glia and neuron concentrations in primary culture. (a)** To determine the number of glia and neurons in primary cultures, we stained all nuclei using Hoechst, and identified neuronal nuclei with a NeuN (Neuronal nucleus) immunostaining. Exemplary images are shown from male and female cultures. Scale bar: 50 µm. **(b)** The dots indicate the number of cells that are NeuN- and/or Hoechst-positive per image, and the graphs show the average ± SEM. The statistical comparison was performed by the Kruskal-Wallis test together with the Dunn’s multiple comparisons test. **(c)** The graph estimates the percentage of neurons in culture, using the data from panel b. The statistical comparison was performed with the unpaired t-test; not significant (ns). **(d)** and **(e)** To investigate the sex-dependent differences in the morphology of the neuronal somas, we analyzed the minor and major axis of NeuN-positive somas. **(f)** Similar to d and e panels, we analyzed the area of the NeuN positive somas. The data are presented as boxplots and whiskers. The boxplots indicate the first and third quartiles and the middle line shows the median. The whiskers show minimum and maximum values. The comparison between the two sexes was performed by the Mann-Whitney test and no significance was found (ns). For all panels, N=3 independent culture preparations, 5 images per condition; not significant (ns).


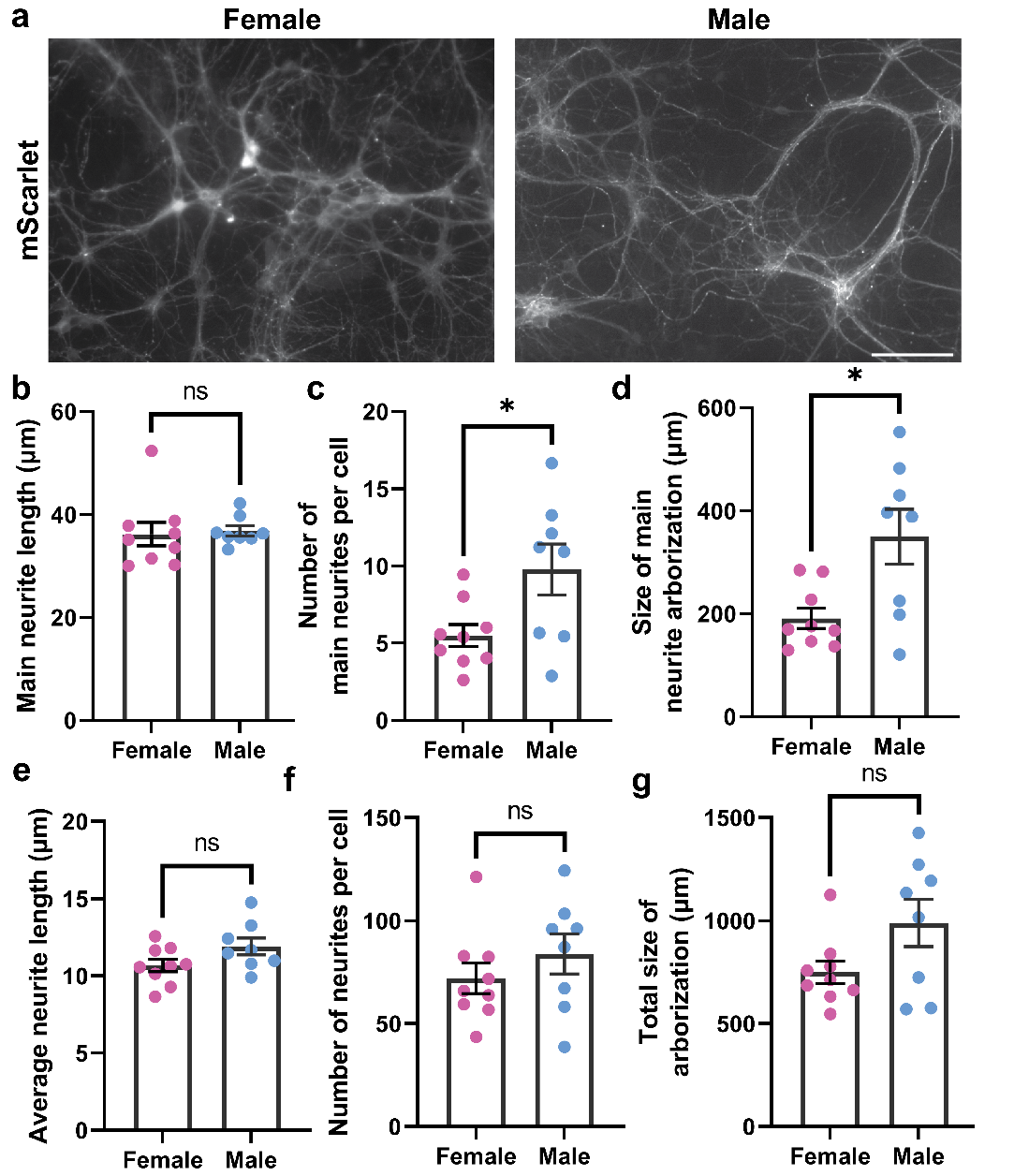


**Supplementary Figure S2. Neuronal volumes in primary hippocampal cultures are similar between female and male neurons. (a)** To estimate the rough volume of neuronal processes, we expressed a membrane-bound Scarlet (mScarlet). Exemplary images for mScarlet-positive female and male neurons are presented. Scale bar: 100 µm. **(b), (c)** and **(d)** To compare the morphology of main (primary) neurites, we calculated the number, length and arborization of intense mScarlet-positive objects, using an empirically-defined threshold that eliminated small objects whose intensity was close to background. **(e), (f)** and **(g)** To compare the morphology of all neurites, we performed a similar analysis to panels b,c and d. We used a different threshold, which enabled us to analyze all mScarlet-positive objects, including small and dim objects. This resulted in an analysis of all detectable neurites. For all panels, dots represent the mean of an image and the bar graph shows the mean ± SEM. N=3 independent culture preparations and 3 large images were taken per condition. The statistical comparison was performed with the unpaired t-test. *p<0.05 and not significant (ns).


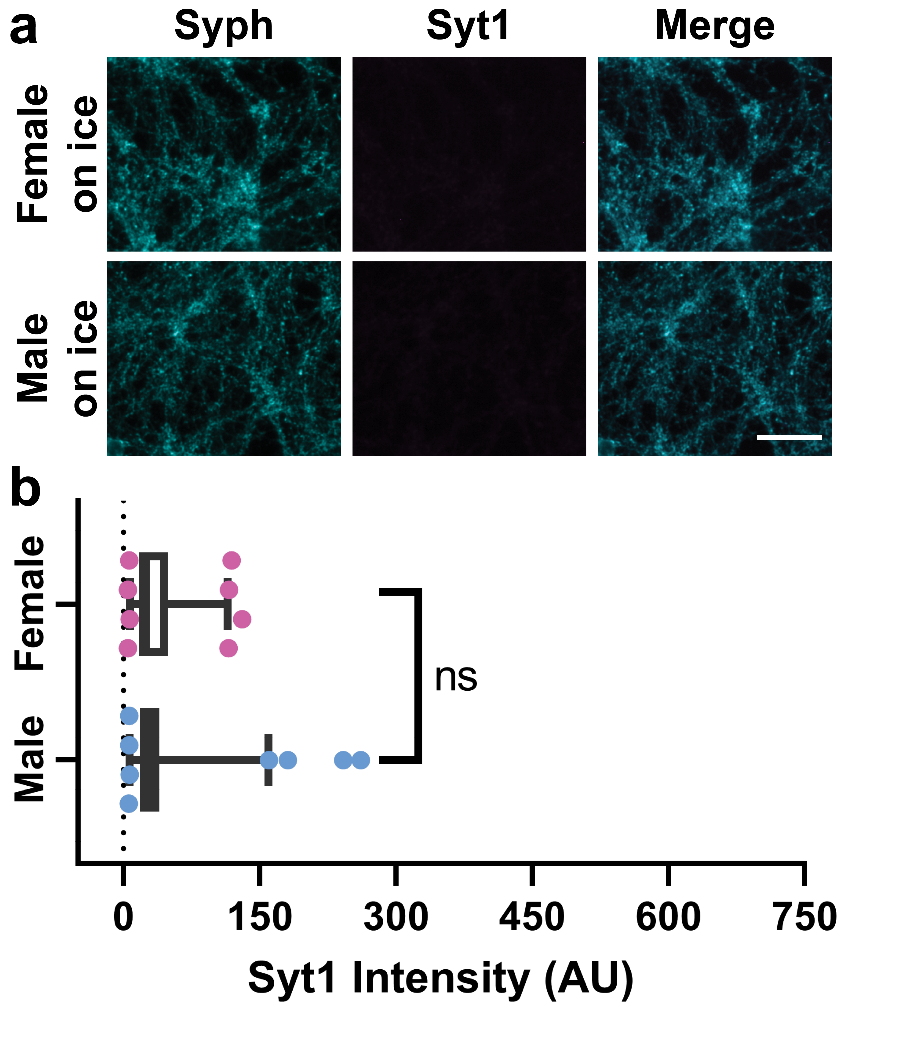


**Supplementary Figure S3. The surface levels of Syt1 are similar in female and male hippocampal cultures. (a)** To estimate the levels of surface Syt1 epitopes, we performed the Syt1 assay (Fig. 1) by incubating the cells on ice. Exemplary images are presented. Scale bar: 50 µm. **(b)** Syt1 intensities in the Syph positive areas were shown in the boxplots represent. The boxplots show the first and third quartiles as well as the median of the distribution. The whiskers indicate the 5-95 percentile. The shown symbols are the outliers and they represent the values obtained from whole images. The statistical comparison between the two sexes was performed by the Mann-Whitney test. not significant (ns). N=3 independent experiments and 5 images per condition.


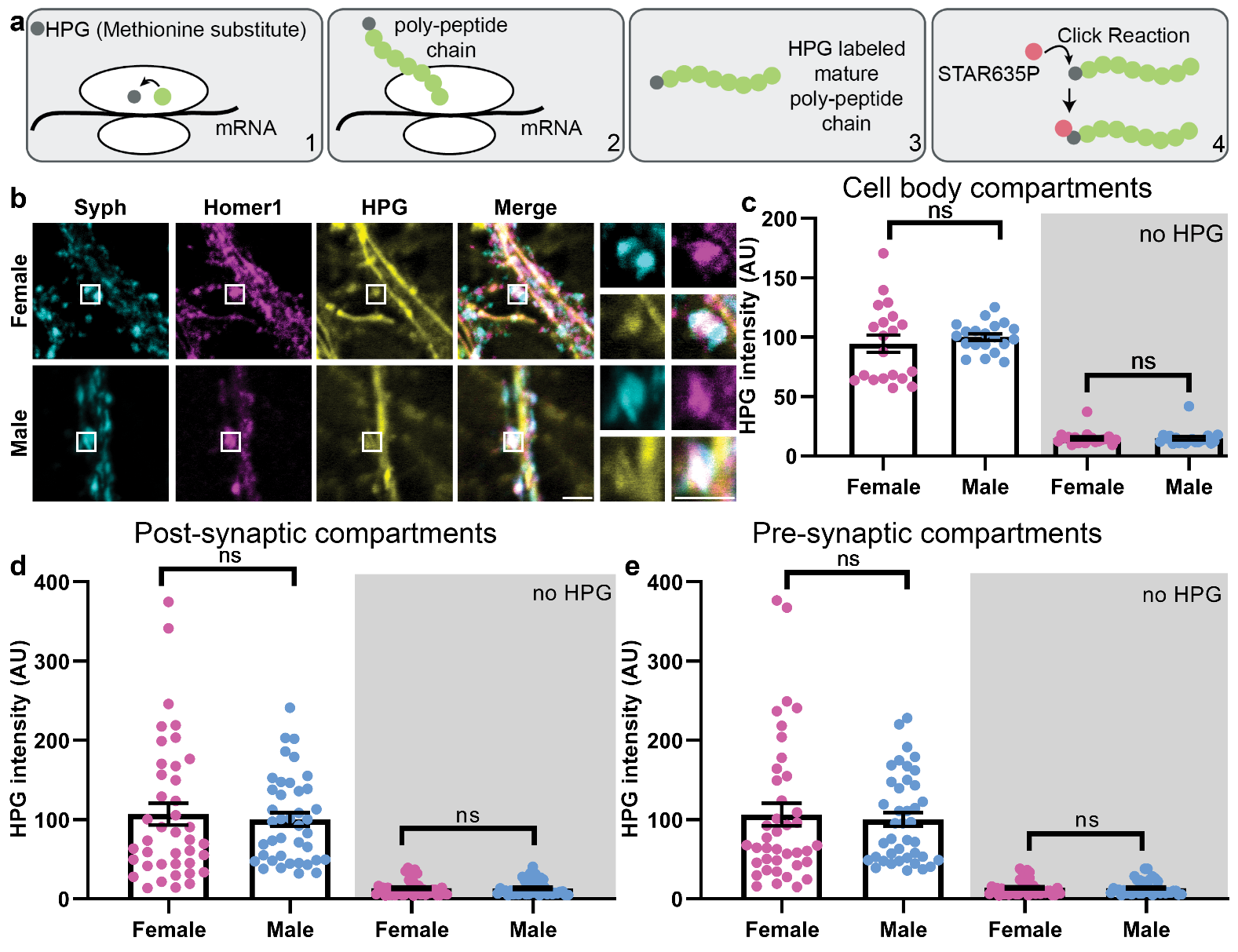


**Supplementary Figure S4. The protein turnover rate at the synapse is not sex-dependent in the primary hippocampal culture. (a)** To investigate the protein turnover rate in the cultures, we performed a metabolic labelling assay using the methionine substitute HPG (1), which is incorporated during translation (2). The metabolic labelling with HPG does not cause major problems in the protein function (3), and enables the labeling of the newly-synthesized proteins, using a click reaction, here using with the fluorophore Star635P (4). To test the specificity of the labeling, we also performed this experiment without the HPG addition (“no HPG”). **(b)** Exemplary images, shown along with Syph and Homer1 immunostainings, to indicate pre- and postsynaptic compartments. Scale bar: 2.5 µm. **(c)**, **(d)**, and **(e)** To determine the protein turnover rate, we calculated the HPG intensity in cell bodies, and in post- and pre-synaptic compartments, respectively. The bar graphs indicate the mean ± SEM. Dots represent the average intensity of an image. The statistical comparison was performed by the Kruskal-Wallis test together with the Dunn’s multiple comparisons test. not significant (ns). N=4 independent culture preparations, 10 images per condition.


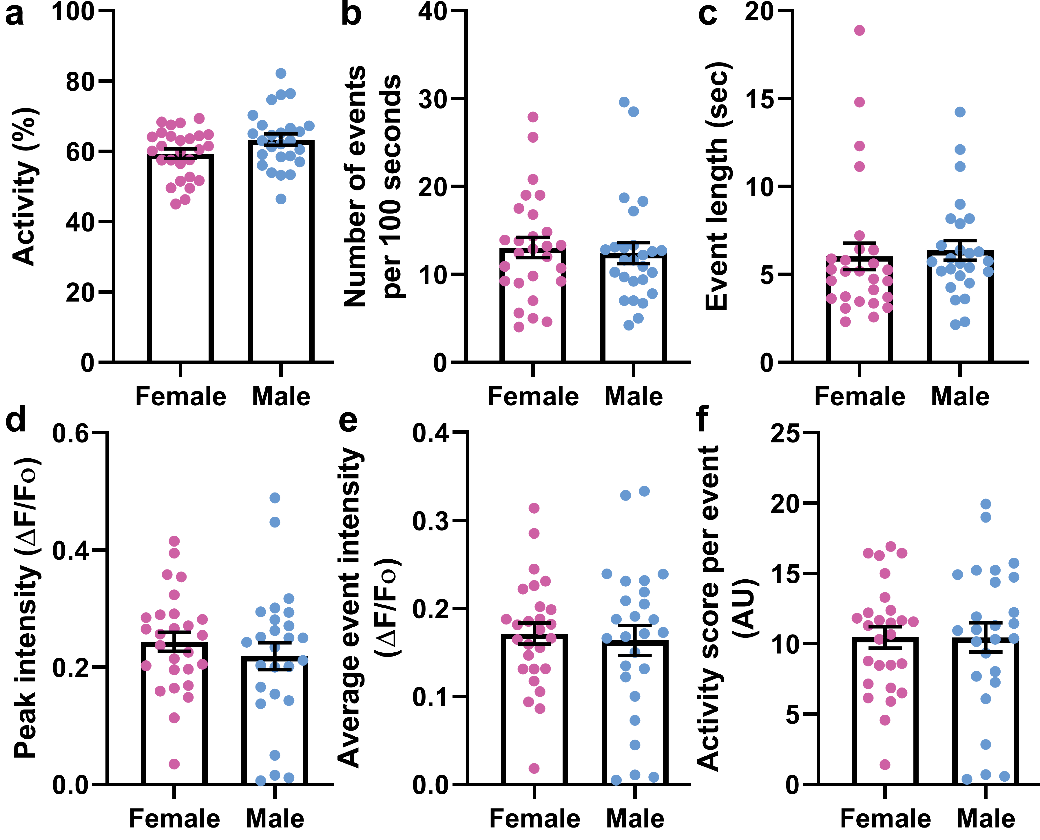


**Supplementary Figure S5. Spontaneous calcium signaling is similar in female and male hippocampal cultures.** In this figure, we analyzed the data that is presented in Fig. 4 in more detail. **(a)** We calculated the % of the time that neurons had an activity above the baseline as a first indication of their levels of activity. **(b)** We calculated the number of events during which the calcium signal rises above the baseline and then decents back to the baseline, over 100 seconds. **(c)** We compared the event length between two sexes. **(d)** We compared the peak instensity between the two sexes. **(e)** Similarly, we compared the average intensity of the events between the two sexes. **(f)** We compared the average activity score per event between female and male neurons. The bar graphs show the mean ± SEM, and each dot represents the mean of a coverslip. The statistical comparison was performed by the Kruskal-Wallis test together with the Dunn’s multiple comparisons test. N=6 independent culture preparations, with up to 6 coverslips per culture. We did not detect any significant difference between the two sexes.


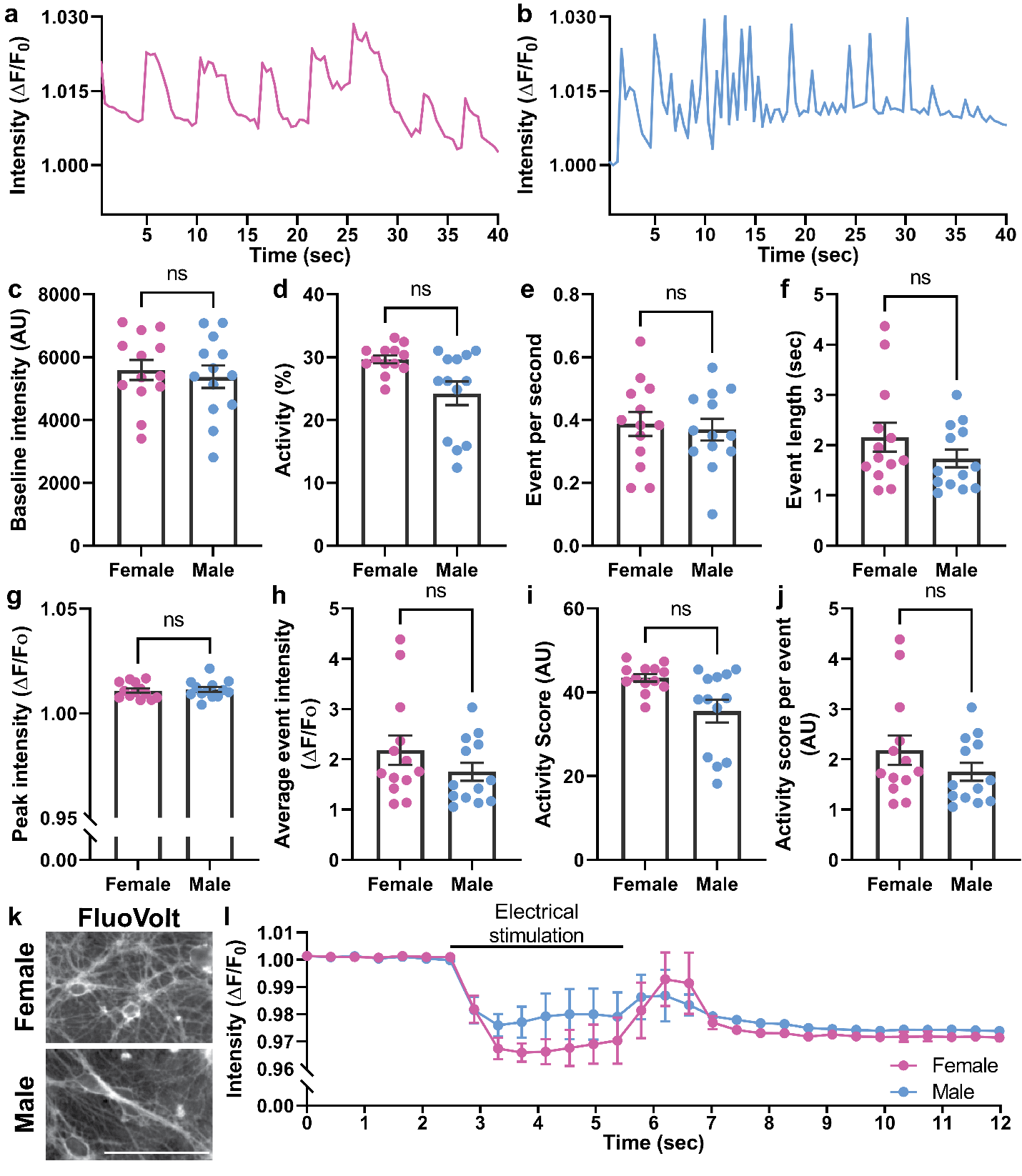


**Supplementary Figure S6. The membrane potential changes do not show any sex-dependent differences under spontaneous or stimulated conditions.** To investigate the sex-dependent differences in membrane potential, we used a voltage-sensitive membrane dye (FluoVolt, Thermo Fisher) and imaged neurons for 5 minutes. We also subjected cultures to 3-second long square pulses (indicated as “electrical stimulation” in panel l). The panels from a-j show analysis before the stimulation, while panels k-l analyze the response of the cultures to the square pulses. **(a)** and **(b)** Exemplary intensity traces from the female and male recordings respectively. **(c)** To test whether the dye uptake shows a difference between female and male cultures, we checked the baseline intensity of all recordings. **(d)** To compare the activity between the two sexes, we calculated percentage for the time that neurons showed an activity above the baseline. **(e)** We analyzed the number of events per second. **(f)** We calculated the duration of each event. **(g)** We compared the peak intensity of each event between the two sexes. **(h)** We compared the average intensity of an event. **(i)** **and** **(j)** As in Fig 4, we calculated the activity score and activity score per event in these recordings. For all panels, N=3 independent cultures, in each culture minimum 4 coverslips were used and during analysis maximum 10 neurons were selected per coverslip. The dots represent the mean of a coverslip, and the bar graph shows the mean±SEM values of both sexes. The statistical comparison was performed by the Kruskal-Wallis test. No significant changes were found (ns). **(k)** Exemplary images for FluoVolt staining in female and male cultures. Scale Bar: 100 µm. **(l)** The intensity traces before, during and after the electrical stimulation in female and male cultures are shown in the graph. The traces were shown with the mean±SEM values of all experiments. The statistical comparison between the two sexes were performed by the Two-way ANOVA test. No significant differences were found.


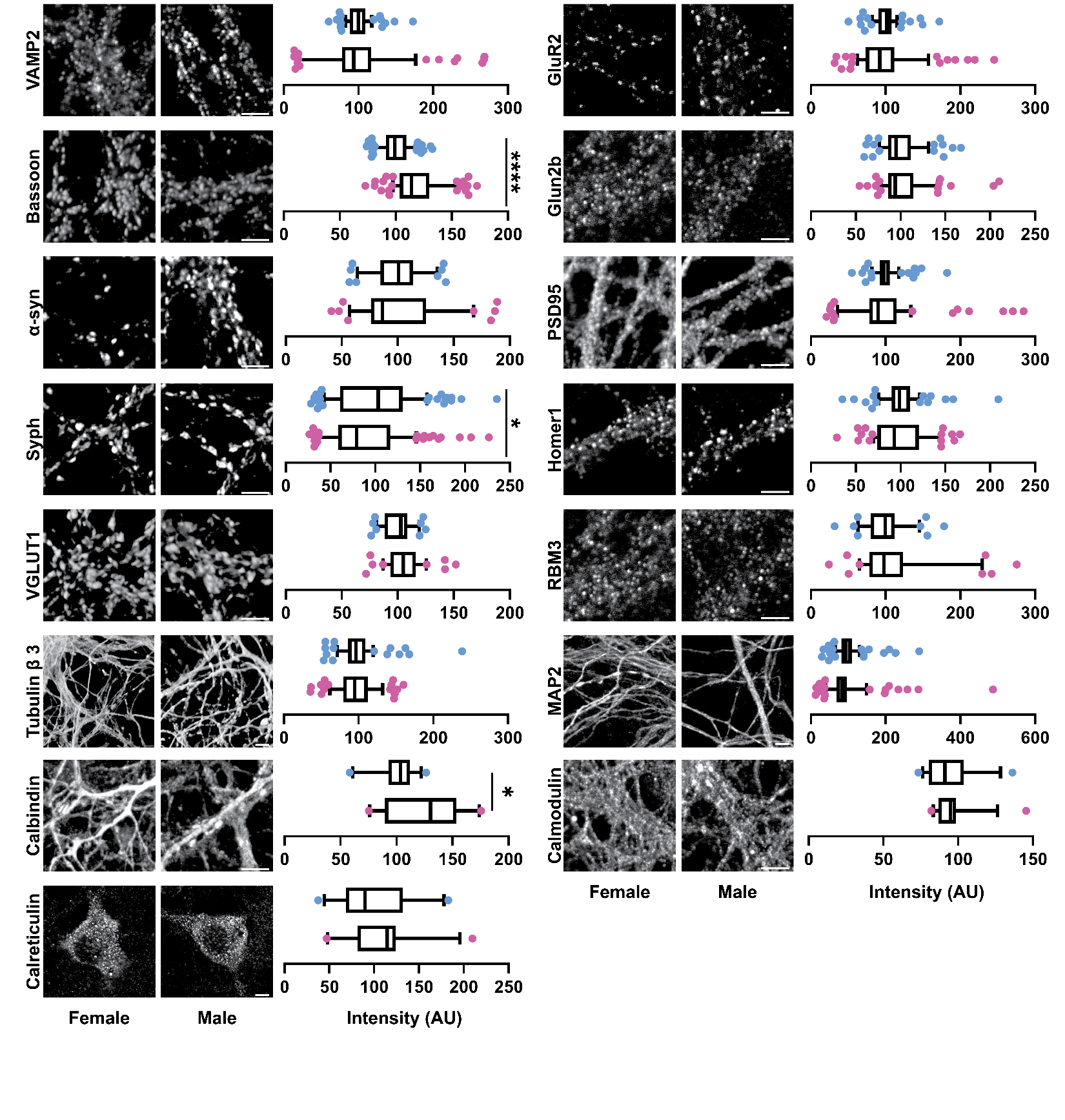
**S****upplementary Figure S7. Immunostainings for multiple synaptic markers are similar for the two sexes.** To determine the differences in synaptic organization between female and male hippocampal neurons in culture, we surveyed the synaptic proteins with immunostainings. Exemplary images for each staining are shown. Scale bar: 3.5 µm. We analyzed the intensity of each staining and plotted these as boxplots. The boxplots indicate the first and third quartiles as well as median of the distribution. The whiskers indicate the 5-95 percentile. The shown symbols are the outliers and they represent the values obtained from whole images. The statistical comparison was done with the Kruskal-Wallis test, and the Dunn’s multiple comparisons test. *p<0.05, ****p<0.0001. N=7 independent culture preparations, except RBM3, which was performed with 4 independent culture preparations, and calcium binding proteins; Calbindin, Calreticulin and Calmodulin, which were performed with 3 independent culture preparations. We found that the synaptic organization is very similar between the two sexes, except for bassoon, synaptophysin and calbindin, for which small, but significant differences could be found.


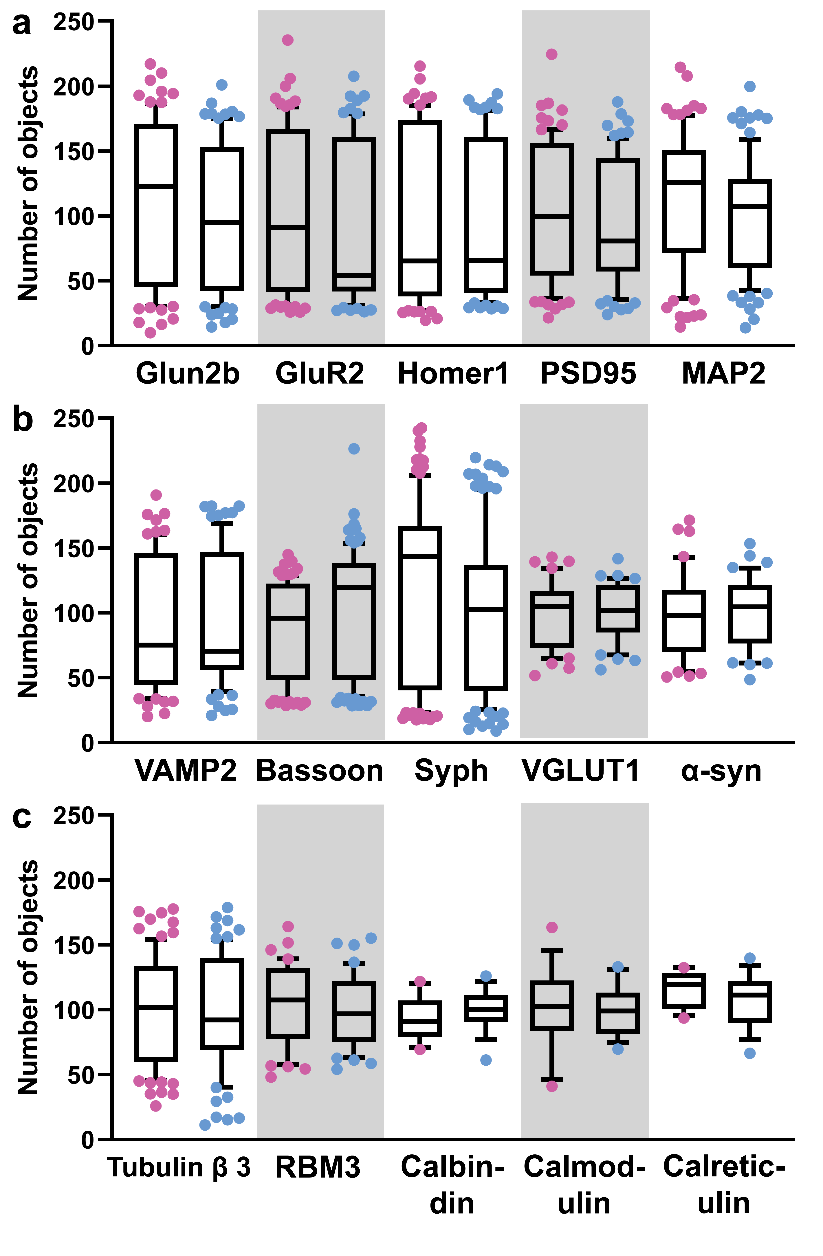


**Supplementary Figure S8. The number of objects detected with our immunostaining survey is very similar between the two sexes.** To determine whether there is a sex-dependent difference in the synapse/object number, we calculated the number of objects for each staining (per image) and compared them in boxplots. **(a)** Post-synaptic markers, **(b)** Pre-synaptic markers and **(c)** we have other stainings as indicated below the graphs. The boxplots indicate the first and third quartiles as well as median of the distribution. The whiskers indicate the 5-95 percentile. The shown symbols are the outliers and they represent the average object numbers in an individual image. The statistical comparison was done by the Kruskal-Wallis test and the Dunn’s multiple comparisons test. We could not observe any significant difference. N=7 independent culture preparations, except RBM3 (N=4), Calbindin (N=3), Calmodulin (N=3) and Calreticulin (N=3).


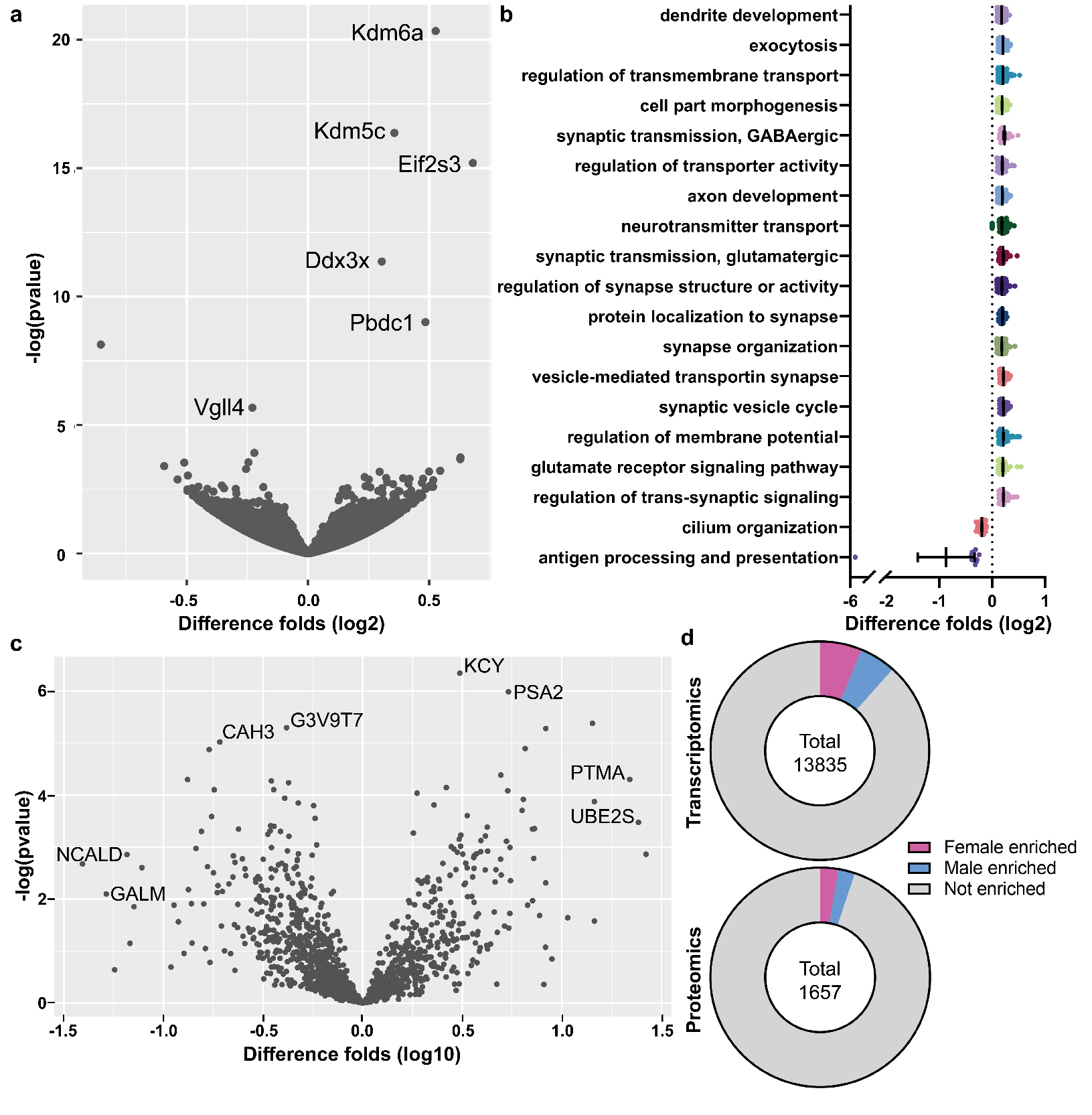
**Supplementary Figure S9. Neuronal activity-related pathways are slightly, but significantly enriched in female hippocampal cultures. (a)** To investigate the differentially expressed mRNAs between female and male hippocampal neurons, we performed mRNA sequencing. The volcano plot displays the log2 fold-changes (x-axis) and the -log10 raw p-values (y-axis) for all detected gene expressions between male and female samples. N=6 independent experiments. **(b)** To determine whether the differentially expressed transcripts enriched in specific pathways, we performed a gene set enrichment analysis (GSEA) with a website called Webgestalt^34^ by clustering the transcripts according to their fold change. The graph shows the mean ± SEM of the fold change of the pathway. Each dot represents a differentially expressed transcript that is part of the pathway. The GSEA analysis calculates that these pathways have FDR (false discovery rate) values which are smaller than 0.05. **(c)** The protein abundances have a similar behavior between female and male hippocampal neurons. To investigate differentially abundant proteins in female and male cultures, we performed a label-free quantitative mass spectrometric approach known as iBAQ, applied to the whole-culture protein lysates. We calculated the female-to-male protein abundance ratio (difference folds) in logarithmic scale 10, and performed statistical testing with the Perseus software. Each dot represents a protein according to its -log(p-value), non-normalized, and to difference folds. N=4 independent culture preparations, and 4 technical replicates. A gene set enrichment analysis (GSEA) was performed as in the panel b, but no significant enrichment was detected. **(d)** The ring graphs show the distribution of transcripts or proteins into the female enriched, male enriched and not enriched categories. The numbers in the middle of the rings indicate the number of transcripts and proteins that are detected with these technologies. The enrichment analysis was described in the a and c panels.

**Supplementary Table S1. The overlap between transcriptomics and proteomics data in terms of female- and male-enriched hits.**

| **Gene symbol** | **Description** | **RNAseq** | **iBAQ** |
| --- | --- | --- | --- |
| Pbxip1 | PBX homeobox interacting protein 1 | 0,89 | 0,514508 |
| Phgdh | phosphoglycerate dehydrogenase | 0,64 | 0,20549 |
| Atic | 5-aminoimidazole-4-carboxamide ribonucleotide formyltransferase/IMP cyclohydrolase | 0,35 | 0,371643 |
| Hnrnpk | heterogeneous nuclear ribonucleoprotein K | 0,29 | 0,123818 |
| Itm2c | integral membrane protein 2C | 0,38 | 0,623169 |
| Rap1b | RAP1B, member of RAS oncogene family | 0,28 | 0,23043 |
| Cct5 | chaperonin containing TCP1 subunit 5 | 0,26 | 0,156549 |
| Eef1d | eukaryotic translation elongation factor 1 delta | 0,41 | 0,210775 |
| Sfxn5 | sideroflexin 5 | 0,75 | 0,603598 |
| Prdx4 | peroxiredoxin 4 | 0,53 | 0,729555 |
| Cstb | cystatin B | 0,33 | -0,33037 |
| Fis1 | fission, mitochondrial 1 | 0,32 | -0,72266 |
| Tmed2 | transmembrane p24 trafficking protein 2 | 0,35 | -0,48741 |
| Psmc4 | proteasome 26S subunit, ATPase 4 | 0,24 | -0,40286 |
| Reep5 | receptor accessory protein 5 | -0,61 | -0,81655 |
| Stxbp1 | syntaxin binding protein 1 | -1,23 | -0,88866 |
| Hnrnpd | heterogeneous nuclear ribonucleoprotein D | -0,91 | -0,20506 |
| Atp1b1 | ATPase Na+/K+ transporting subunit beta 1 | -0,52 | 0,248165 |
| Psma3 | proteasome subunit alpha 3 | -0,34 | 0,257176 |
| Uso1 | USO1 vesicle transport factor | -0,24 | 0,461812 |
| Tuba1a | tubulin, alpha 1A | -0,51 | 0,364783 |
| Wasf1 | WAS protein family, member 1 | -2,46 | 0,508245 |
| Rtn3 | reticulon 3 | -0,9 | 0,510715 |
| Rac1 | ras-related C3 botulinum toxin substrate 1 | -0,22 | 0,259821 |

**Supplementary Methods**

**Transfection.** Cultures were transfected at an early age (DIV5) using Lipofectamine 2000 (Thermo Fisher, US), according to the manufecturer’s protocol. The pUC57 plasmid contains a ubiquitin C (UBC) promoter and a membrane-bound Scarlet (mScarlet). At DIV15 coverslips were immunostained against mScarlet protein (see the Supplementary Table 2), as described in the immunostaining section.

**Image analysis for morphology.** The number of neuron and glia cells were calculated relying on Hoechst labeling (for all cells) and NeuN immunostaining (for neurons). The morphological properties of somas were analyzed from NeuN stainings. After applying an intensity threshold, the minor and major axis, as well as the area of these objects, were calculated. Similarly, mScarlet labels were subjected to different intensity thresholds, to reveal large, bright objects (labeled as ‘main neurites’) or all objects (labeled as ‘all neurites’). Neurite length was then measured and compared among the different cultures. The neurite numbers and the total arborization (summing neurite length for all neurons in one image) was then measured and compared between female and male cultures. All the analysis was performed with Matlab (The MathWorks Inc., Natick, MA, USA).

**Supplementary Table S2.** The primary and secondary antibodies that are used in this study.

| Antibody name | Company | Catalog No. | Ratio |
| --- | --- | --- | --- |
| VAMP2 | Synaptic Systems | 104 202 | 1:500 |
| Bassoon | Enzo | ADI-VAM-PS003-F | 1:500 |
| Alpha-synuclein | BD Biosciences | 680787 | 1:500 |
| Synaptophysin | Synaptic Systems | 101 004 | 1:500 |
| VGLUT1 | NanoTag | N1602-Ab580-L | 1:500 |
| VGAT | Synaptic Systems | 131 103 | 1:250 |
| Homer1 | Synaptic Systems | 160 011 | 1:500 |
| GluR2 | Alomone Lab | AGC-005 | 1:500 |
| Glun2b | NeuroMab | 75-101 | 1:500 |
| PSD95 | Cell Signaling | 3450 | 1:500 |
| MAP2 | Synaptic Systems | 188 002 | 1:250 |
| Tubulin ß3 | BioLegend Company | PRB-435P-100 | 1:300 |
| Calbindin D28K | Synaptic Systems | 214 002 | 1:300 |
| Calreticulin | Cell Signaling | 122 238 | 1:250 |
| Calmodulin | Abcam | Ab45689 | 1:600 |
| RBM3 | Abcam | Ab134946 | 1:250 |
| Puromycin | MERCK Millipore | MABE343 | 1:500 |
| mScarlet NB | Nanotag | N1302-At565-S | 1:250 |
| NeuN | Synaptic Systems | 266 011 | 1:250 |
| Alexa 488 (anti-mouse ) | Dianova | 715-545-151 | 1:200 |
| Alexa 488 (anti-guinea pig) | Dianova | 706-545-148 | 1:200 |
| Alexa 488 (anti-chicken) | Dianova | 703-545-155 | 1:200 |
| Cy3 (anti-guinea pig) | Dianova | 706-165-148 | 1:200 |
| Cy2 (anti-chicken) | Dianova | 103-225-155 | 1:200 |
| Star 635P (anti-mouse) | Abberior | ST635P-1001-500µg | 1:200 |
| Star 635P (anti-rabbit) | Abberior | ST635P-1002-500UG | 1:200 |

**Membrane potential imaging.** FluoVolt (Thermo Fisher, US) was used as a voltage-sensitive membrane dye, following the manufacturer’s instructions (2.5 µl of the Component A was mixed with 25 µl of the Component B). On DIV18 coverslips were incubated with 400 µl of the Tyrode together with 4 µl of the FluoVolt mixture for 30 min in the incubator. After the incubation, coverslips were washed with room temperature Tyrode and imaged at 37°C with an inverted Nikon Ti eclipse epifluorescence microscope (Nikon, Japan), as for the calcium imaging (see description in the Methods section in the main text). Coverslips were imaged for 5 min. At the 90^th^ second, coverslips were subject to a square pulse of 3 sec in length. We selected up to 10 ROIs, near the somas, in every movie and then analyzed the average intensity in each frame (depicted in the respective figure). All other analyses of these signals were performed as described for the calcium imaging.

**FUNCAT (Fluorescent Non-Canonical Amino Acid Tagging) assay.** 0.2 mM HPG (4-Hydroxyphenylglycine, C10186, Thermo Fisher, US) was added into the 300 µl of DMEM which is supplemented with 6.5 mM HEPES (Invitrogen, US), B27 supplement (Thermo Fisher, US), 0.25 mM L-cysteine (Invtirogen, US) and 0.81 mM MgCl_2_, and incubated for 4 hours. After washing with Tyrode buffer, cells were fixed with 4% PFA. Star635P-azide (Abberior, Germany) was conjugated to HPG, with a click reaction (Click-iT reaction buffer kit, Thermo Fisher, US), following manufacturer’s protocol. To determine the background signal, we did not add any HPG in the media of the control group. As synaptic markers, Homer1 and Syph proteins were used and immunostaining protocols are described in the immunostaining section.

**Transcriptomics.** RNA was isolated from the culture with the miRNeasy Kit (Qiagen, France). The mRNA sequencing and primary analysis were performed by the NGS Integrative Genomics Core Unit (NIG, Göttingen, Germany). In brief, sequenced reads were aligned to the Rattus Norvegicus genome assembly rn6 from ENSEMBL (<https://www.ensembl.org/Rattus_norvegicus/Info/Index>) using STAR aligner version 2.5.2a^31^ allowing for 2 mismatches within 50 bases. The uniquely mapped reads were subsequently quantified for all genes in Rattus Norvegicus genome assembly rn6 version 100 using featureCounts version v1.5.0-p1^32^. The R package DESeq2 version 1.29.4 was used for differential expression analysis^33^, where differentially expressed genes were defined as having an adjusted p-value <=0.05 and an absolute log2 fold-change >=1. Using Ensemble gene IDs of differentially expressed transcripts, Webgestalt database was used for gene set enrichment analysis of KEGG pathwaysusing the fold changes between the two sexes as the rank score^34^. The result of GSEA (gene set enrichment analysis) indicates the pathways that have a p-value lower than 0.05 and a false-discovery rate (FDR) lower than 0.05. The transcripts with significant differences between the sexes are listed in the Supplementary Fig. 8.

**miRNA-omics.** miRNA was isolated from the culture with the miReasy Kit (Qiagen, France). The miRNA-seq sequencing and primary analysis were performed by the NGS Integrative Genomics Core Unit (NIG, Göttingen, Germany). In brief, sequenced reads were initially trimmed for Illumina Small RNA 3’ Adapter using cutadapt version 2.10^35^. The trimmed reads were aligned to the Rattus Norvegicus non-coding regions in rn6 from ENSEMBL (<https://www.ensembl.org/Rattus_norvegicus/Info/Index>) using bowtie2 version 2.3.4^36^ with default parameters. High-quality mapped reads (MAPQ=1 or MAPQ>4) were selected from the resulting alignment files and quantified for the non-coding regions in Rattus Norvegicus genome assembly rn6 using Salmon version 1.2.1^37^ using traditional expectation maximization (EM) algorithm.

**iBAQ sample processing.** The protein lysate was collected by scraping cultures with 100 μl of Tyrode buffer. The protein estimation was performed with the standard BCA protocol^38^, as provided by the manufacturer (Thermo Scientific, US). 20 ug of protein sample was heated to 95°C for 5 min with 10 μl of 1% RapiGest. The following steps were performed at 750 rpm on a thermomixer at the room temperature. Samples were incubated for 5 min with 10 μl of 100 mM ammonium bicarbonate solution and later with 10 μl of 10 mM dithiothreitol in 100 mM ammonium bicarbonate for 1 hour, to reduce cysteines. For alkylation of reduced cysteines, samples were incubated for 20 min in dark with 10 μl of 100 mM iodoacetamide in 100 mM ammonium bicarbonate. To reduce the detergent concentration to 0.1%, 180 μl of 100 mM ammonium bicarbonate was added into samples. Finally, for digestion trypsin with 1:20 ratio (ProMega, US) was added. Samples were treated with 20 μl of 5% formic acid solution to quench the trypsin activity. To deteriorate the detergent, 20 μl of 5% trifluoro-acetic acid was added and incubated for 2 hours. StageTips was used for desalting the samples. Briefly, to make one column at least four C_18_ plugs were filled in a micropipette tip. The column washing was done twice with 50 μl of methanol and it was equilibrated twice with 0.1% formic acid. The peptides in supernatant were loaded on a pre-equilibriated column. While passing the supernatant through column, the peptides being hydrophobic bound to the C_18_ matrix. 50 μl of 0.1% formic acid was used to wash the column twice. Lastly, the elution of bound peptides were done twice with a solution (50 μl of 80% acetonitrile, 0.1% formic acid solution). SpeedVac is used to dry the peptide solution. 1 μg of digested protein sample (male or female or UPS2 standard protein) were injected and processed for LC-MS on a 90 min gradient on Q-Exactive HF Mass Spectrometer (Thermo Scientific, US). UPS2 standard protein was run in between the samples.

**Liquid chromatography mass spectrometry (LC-MS).** Peptides were resuspended in the sample loading buffer (5% acetonitrile and 0.1% trifluoroactic acid). Later, they were fractionated. The analysis was performed with an online UltiMate 3000 RSLCnano HPLC system (Thermo Fisher Scientific, US) that is coupled to the Q Exactive HF or Orbitrap Fusion Lumos Tribrid Mass Spectrometer (Thermo Fisher, US). For 3 minutes, salts were removed from peptides with a reverse phase C_18_ pre-column (3 cm long, 100μm inner diameter 360 mm outer diameter) and it was exchanged with an analytical column (30 cm long, 75 μm inner diameter) which is prepared in-house using ReproSil-Pur C_18_ AQ 1.9 μm reversed phase resin (Dr. Maisch GmbH). The separation of peptides were performed with a linear gradient of 5–30% buffer B (80% acetonitrile (Lichrosolv, (Merck Millipore, US)) together with 0.1% formic acid) at flow rate of 10 nL/min gradient of 88 min on Q-Exactive HF. The temperature of the columns were set to 50°C. The scanning mass range is: 350 to 1600 Da and the resolution is: 60,000 at m/z 200. For MS1, only the top 30 precursor ions were selected and they were subjected to HCD fragmentation in MS2 with 1e5 ions of the AGC target fill value. The window for precursor isolation was set to 1.4 Da. For internal recalibration, the lock mass option was set as m/z 445.1200^39^ .

**Data analysis for iBAQ.** For identifying the proteins: MaxQuant software^40^ version 1.5.3.8 or 1.6.0.16, the Andromeda search engine^41^ with rat SwissProt (December 2016; containing 29795 entries) and Human Universal Proteome Standard (UPS2, Sigma-Aldrich) protein databases. During the search, tolerance for MS was set as 6 ppm and for MS/MS as 10 ppm. Carbamidomethylation of cysteines and oxidation of methionine were set as fixed and variable modifications respectively. For tryptic specificity, there was no proline restriction and maximum 2 missed cleavages were allowed. Up to 1% False discovery rate (FDR) was accepted. Moreover, for quantification the LFQ and iBAQ option were used. Perseus was used for further data analysis^42^.

**Supplementary information.** The transcriptomics and proteomics data are available as an excel sheet.
